# Structural insights into G protein-coupled receptor signaling

**DOI:** 10.1063/4.0000998

**Published:** 2025-10-27

**Authors:** Andrew Kruse

**Affiliations:** Harvard Medical School, Boston, MA, USA

## Abstract

G protein-coupled receptors (GPCRs) are the largest single family of transmembrane proteins encoded in the human genome, and they are among the most successful classes of therapeutic drug targets of all time. Signaling by GPCRs relies on an intricate relay of conformational changes that can be induced, stabilized, or prevented by ligands. Using the angiotensin II type 1 receptor (AT1R) and the RXFP1 relaxin receptor as model systems, we investigated how protein ligands and small molecule drugs differentially regulate receptor activation and signaling outputs. In the case of AT1R we find that antibody fragments can selectively stabilize unique conformations and act as either competitive or allosteric inhibitors. In the case of RXFP1, we find that a small molecule drug candidate induces distinct conformational changes and different signaling outputs than the native ligand relaxin-2. Collectively, these results shed light on shared and divergent aspects of GPCR conformational regulation by protein and small molecule binders, with implications for next-generation GPCR-targeted drug discovery efforts.

